# Estimated glomerular filtration rate by serum creatinine or standardized cystatin C in Japanese patients with Graves׳ disease

**DOI:** 10.1016/j.dib.2015.11.023

**Published:** 2015-11-24

**Authors:** Yoshitake Suzuki, Kazuyuki Matsushita, Masanori Seimiya, Toshihiko Yoshida, Yuji Sawabe, Makoto Ogawa, Fumio Nomura

**Affiliations:** a Department of Laboratory Medicine, Chiba University Hospital, 1-8-1 Inohana, Chuo-ku, Chiba, Chiba 260-8677, Japan; b Department of Molecular Diagnosis, Chiba University Graduate School of Medicine, 1-8-1 Inohana, Chuo-ku, Chiba, Chiba 260-8677, Japan; c Department of Gastroenterology and Nephrology, Chiba University Graduate School of Medicine, 1-8-1 Inohana, Chuo-ku, Chiba, Chiba 260-8677, Japan

**Keywords:** sCysC, serum cystatin C, sCr, serum creatinine, eGFR, estimated glomerular filtration rate, eGFR_Cr_, estimated glomerular filtration rate calculated by serum creatinine, eGFR_Cys_C, estimated glomerular filtration rate calculated by standardized serum cystatin C, TSH, thyroid stimulating hormone, FT3, free triiodothyronine, FT4, free thyroxine, GD, Graves׳ disease, Standardized cystatin C, Creatinine, Estimated glomerular filtration rate, Thyroid hormone

## Abstract

Glomerular filtration rate (eGFR) by serum creatinine (eGFR_Cr_) or standardized cystatin C (eGFR_CysC_) were estimated in Japanese patients with Graves׳ disease (GD) of different sex. Clinical samples were collected from patients with GD with normal renal function to accurately validate eGFR_Cr_ and eGFR_CysC_ levels and evaluate how hyperthyroidism affects renal function. Levels of eGFR_Cr_ and eGFR_CysC_ showed clinical usefulness in successfully treated euthyroid patients with GD regardless of sex. The article includes detailed experimental methods and data used in our analysis. The data relates to the “Paradoxical effect of thyroid function on the estimated glomerular filtration rate by serum creatinine or standardized cystatin C in Japanese Graves’ disease patients” (Suzuki et al., 2015) [1]

**Specifications Table**

**Table:** 

Subject area	Biochemistry
More specific subject area	Renal function
Type of data	Methods, figures, and tables
How data were acquired	Auto-analyzer
Data format	Analyzed
Experimental factors	Control and disease patients in remission or not
Experimental features	Correlation between elevated thyroid hormones and estimated glomerular filtration rate, and effecting analyses before and after treatment in remission and non-remission groups of serum samples collected from patients with Graves׳ disease
Data source location	Chiba University Hospital in Japan
Data accessibility	All publicly released data are within this article

## Value of the data

• The data included important resources for clinician and researchers regarding changes in estimated glomerular filtration rate (eGFR) levels when evaluating renal function in patients with Graves׳ disease (GD) in the field of clinical medicine.
• The article provides analysis data of two different eGFR equations depending to sex.
• Tha data showing effect of treatment in patients with GD is useful for other researchers and clinicians.

## 1. Experimental design and data

GD is an autoimmune disease that affects thyroid function. It frequently results in hyperthyroidism and an enlarged thyroid gland. Typically, increased free triiodothyronine (FT3), free thyroxine (FT4), and thyroid stimulating hormone (TSH) antibodies and decreased TSH are noted in clinical examination. GD occurs in approximately 0.5% of patients [2] and is approximately 7.5 times more frequent in women than men [2]. We collected 113 outpatients with untreated or poorly controlled GD. Control subjects included 146 healthy volunteers without physical or clinical abnormalities upon routine health check. Clinical characteristics of clinical samples from males and females have been described in a related report [1]. The patients with GD were categorized into two groups (remission or non-remission), following pharmacotherapy indicated by the Japan Thyroid Association guidelines (methimazole and/or propylthiouracil). Diagnostic criteria were based on the changes in TSH, FT3, and FT4 levels following treatment [1]. Clinical characteristics have been described in the accompanying report before and after treatment during the medication period (8.2±2.8 months) in the remission and non-remission groups [1]. The changes in TSH, FT3, and FT4 levels, before and after treatment, in the remission and non-remission groups are listed in Supplemental Fig. 1. Remission group returned to reference interval (TSH: <0.003 to 2.02±0.85, FT3: 14.4±10.1 to 4.2±0.7, FT4: 29.0±12.2 to 11.7±2.3) and non-remission group did not returned to reference interval (TSH: <0.003 to 0.016, FT3: 18.9±12.2 to 8.5±5.5, FT4: 32.5±11.3 to 21.0±7.5). Correlations between eGFR calculated by serum creatinine (sCr) levels (eGFR_Cr_) and elevated FT3 and FT4 levels in males or females with GD are shown in Supplemental Fig. 2. eGFR_Cr_ levels of males (bias: 2.42, 1.81) and females (bias: 2.14, 1.86) are increasing with elevated FT3 and FT4 levels. Correlations between eGFR calculated by standardized serum cystatin C (sCysC) levels (eGFR_CysC_) and elevated FT3 and FT4 levels in males and females with GD are shown in Supplemental Fig. 3. eGFR_CysC_ levels of males (bias: −0.86, −0.72) and females (bias: −0.88, −0.72) are decreasing with elevated FT3 and FT4 levels. Validation of the effect on levels of eGFR_Cr_ and eGFR_CysC_, before and after treatment, in remission and non-remission groups are shown in Supplemental Fig. 4. Levels of eGFR_Cr_ and eGFR_CysC_ after treatment in remission group showed same tendency (102.8±7.4 and 105.6±8.7), but levels of eGFR_Cr_ and eGFR_CysC_ before treatment in remission, before and after in non-remission groups showed different tendency (138.7±25.3 and 75.9±12.9, 136.2±33.6 and 74.8±19.1, 120.2±27.7 and 85.4±19.6).

## 2. Materials and methods

### 2.1 Preparation of biological samples

Blood samples were obtained using vacuum blood collection tubes with coated glass powder to prevent blood coagulation (Kyokuto Seiyaku, Tokyo, Japan) in the blood-drawing room at Chiba university hospital. After coagulation, blood samples were centrifuged (Hitachi, Tokyo, Japan) at 3000 g for 7 min, and hemocytic components and serum were separated. Serum samples were stored at −80 °C until further use. Written informed consent was obtained from all patients and healthy control subjects before blood sampling. Our study protocol was approved by the ethics committee of Chiba University Graduate School of Medicine.

### 2.2 Data analysis

The based data used in this study were obtained as follows: sCr levels were measured using an enzymatic method (CRE LM; Wako, Osaka, Japan), with a reference interval of 54–92 μmol/L and 42–70 μmol/L for males and females, respectively, and a coefficient of variation (CV) of 1.0%. The procedure for measuring sCysC was standardized based on international reference material published in 2010 [3]. Standardized sCysC levels were measured using latex turbidimetric immunoassay (N-assay LA cystatin C; Nittobo, Tokyo, Japan), with a reference interval of 0.60–0.98 mg/L and 0.49–0.82 mg/L for males and females, respectively, and a 0.9% CV. Standardized sCysC and sCr were measured using a BioMajesty 8040 analyzer (JEOL, Tokyo, Japan) according to the manufacturer׳s instructions. Serum TSH, FT3, and FT4 levels were measured by chemiluminescent immunoassay using an ARCHITECT i2000SR analyzer (Abotto Japan, Tokyo, Japan) according to the manufacturer׳s instructions. Reference intervals were as follows: TSH, 0.35–4.94 mIU/L (CV=1.6%); FT3, 2.6–5.7 pmol/L (CV=9.8%); and FT4, 9.0–19.1 pmol/L (CV=9.9%).

### 2.3 Calculation of eGFR

In this report, renal function was evaluated using eGFR_Cr_ and eGFR_CysC_, which were calculated using equations reported by the Japanese Society of Nephrology as follows [3], [4]:

eGFR_Cr_=194×(sCr×0.011312)^−1.094^×(Age)^−0.287^ (×0.739 if female)

eGFR_CysC_=(104×(standardized sCysC)^−1.019^×Age^0.996^ (×0.929 if female))−8

eGFR_Cr_ and eGFR_CysC_ are expressed as mL/min/1.73 m^2^ of body surface area. sCr is expressed as mg/dL (if μmol/L/88.6), and standardized sCysC is expressed as mg/L [3], [4].

### 2.4 Statistical analysis

Summary statistics were constructed using frequencies and proportions for categorical data and means and standard deviations (SD) for continuous variables. Student׳s t-test was used to compare groups in all experiments. All comparisons were planned, and statistical tests were two-sided. All statistical analyses were performed using StatFlex version 5.0 (ArTeC Inc., Osaka, Japan). Mean and SD were used for descriptive statistics. *p* values<0.05 were considered statistically significant.

## Appendix A Supplementary material

Supplementary material

Supplemental Fig. 1 Comparison of TSH, FT3, and FT4 levels in remission and non-remission groups, following pharmacotherapy (methimazole or propylthiouracil) in patients with untreated or poorly controlled GD. TSH, FT3, and FT4 are indicated by diamonds (◇). (a) TSH levels before and after treatment (remission group), (b) FT3 levels before and after treatment (remission group), (c) FT4 levels before and after treatment (remission group), (d) TSH levels before and after treatment (non-remission group), (e) FT3 levels before and after treatment (non-remission group) and (f) FT4 levels before and after treatment (non-remission group).

Supplemental Fig. 2 Relationships between serum thyroid hormone levels (FT3 and FT4) and eGFR_Cr_ levels in males and females with GD. Estimated glomerular filtration rates (eGFR) were determined from serum creatinine (sCr) levels (eGFR_Cr_) in patients with untreated or poorly controlled GD (*n*=113). eGFR_Cr_ levels in male patients are indicated by circles (○) and in female patients by triangle (△). (a) Relationship between free triiodothyronine (FT3) levels (*x*-axis) and eGFR_Cr_ levels (*y*-axis) in male patients, (b) relationship FT3 levels (*x*-axis) and eGFR_Cr_ levels (*y*-axis) in female patients, (c) relationship between free thyroxine (FT4) levels (*x*-axis) and eGFR_Cr_ levels (*y*-axis) in male patients and (d) relationship between FT4 levels (*x*-axis) and eGFR_Cr_ levels (*y*-axis) in female patients.

Supplemental Fig. 3 Relationships between serum thyroid hormone levels (FT3 and FT4) and eGFR_CysC_ levels in males and females with GD. Estimated glomerular filtration rates (eGFR) were determined from standardized serum cystatin C (sCysC) levels (eGFR_CysC_) in patients with untreated or poorly controlled GD (*n*=113). eGFR_CysC_ levels in male patients are indicated by circles (○) and in female patients by triangle (△). (a) Relationship between free triiodothyronine (FT3) levels (*x*-axis) and eGFR_CysC_ levels (*y*-axis) in male patients, (b) relationship FT3 levels (*x*-axis) and eGFR_CysC_ levels (*y*-axis) in female patients, (c) relationship between free thyroxine (FT4) levels (*x*-axis) and eGFR_CysC_ levels (*y*-axis) in male patients and (d) relationship between FT4 levels (*x*-axis) and eGFR_CysC_ levels (*y*-axis) in female patients.

Supplemental Fig. 4 Evaluation of eGFR_Cr_ and eGFR_CysC_ levels in patients with GD, before and after treatment, in remission and non-remission groups. Estimated glomerular filtration rates (eGFRs) were determined from serum creatinine (sCr) levels (eGFR_Cr_) and standardized serum cystatin C (sCysC) levels (eGFR_CysC_) in remission (*n*=41) and non-remission (*n*=72) groups. eGFRs are indicated by diamonds (◇). (a) Levels of eGFR_Cr_ and eGFR_CysC_ before treatment (remission group), (b) levels of eGFR_Cr_ and eGFR_CysC_ after treatment (remission group), (c) levels of eGFR_Cr_ and eGFR_CysC_ before treatment (non-remission group) and (d) levels of eGFR_Cr_ and eGFR_CysC_ after treatment (non-remission group).

## Footnotes

Appendix A Supplementary data associated with this article can be found in the online version at doi:10.1016/j.dib.2015.11.023.
