# Supplementary figures and images for: Estimated glomerular filtration rate by serum creatinine or standardized cystatin C in Japanese patients with Graves׳ disease
Source: Data Brief. 2015 Nov 24;5:1092–5. doi: 10.1016/j.dib.2015.11.023 (PMC4689117; doi:10.1016/j.dib.2015.11.023)

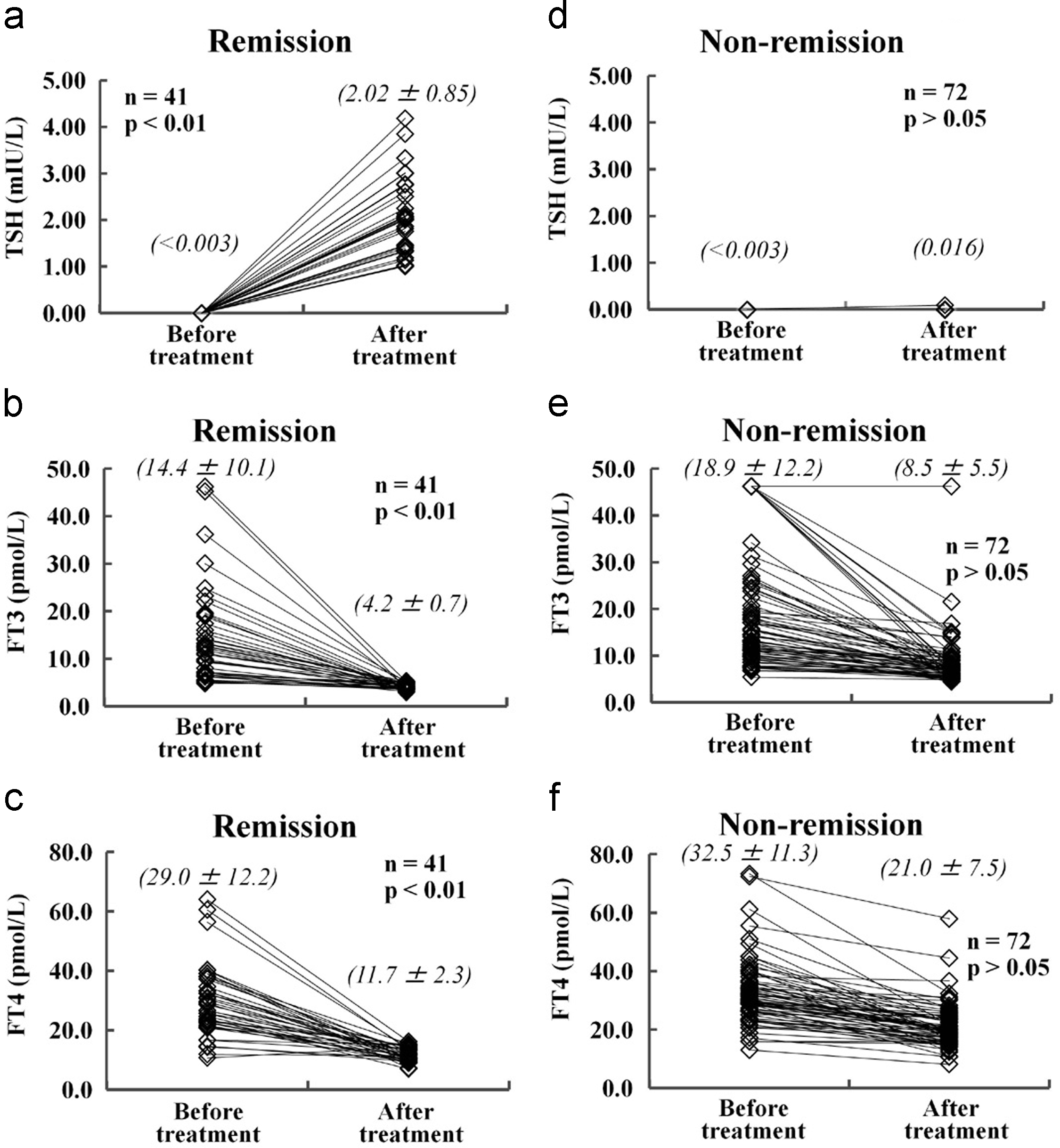

Supplement: Supplemental Fig. 1 — Comparison of TSH, FT3, and FT4 levels in remission and non-remission groups, following pharmacotherapy (methimazole or propylthiouracil) in patients with untreated or poorly controlled GD. TSH, FT3, and FT4 are indicated by diamonds (◇). (a) TSH levels before and after treatment (remission group), (b) FT3 levels before and after treatment (remission group), (c) FT4 levels before and after treatment (remission group), (d) TSH levels before and after treatment (non-remission group), (e) FT3 levels before and after treatment (non-remission group) and (f) FT4 levels before and after treatment (non-remission group). [file mmc2.jpg]

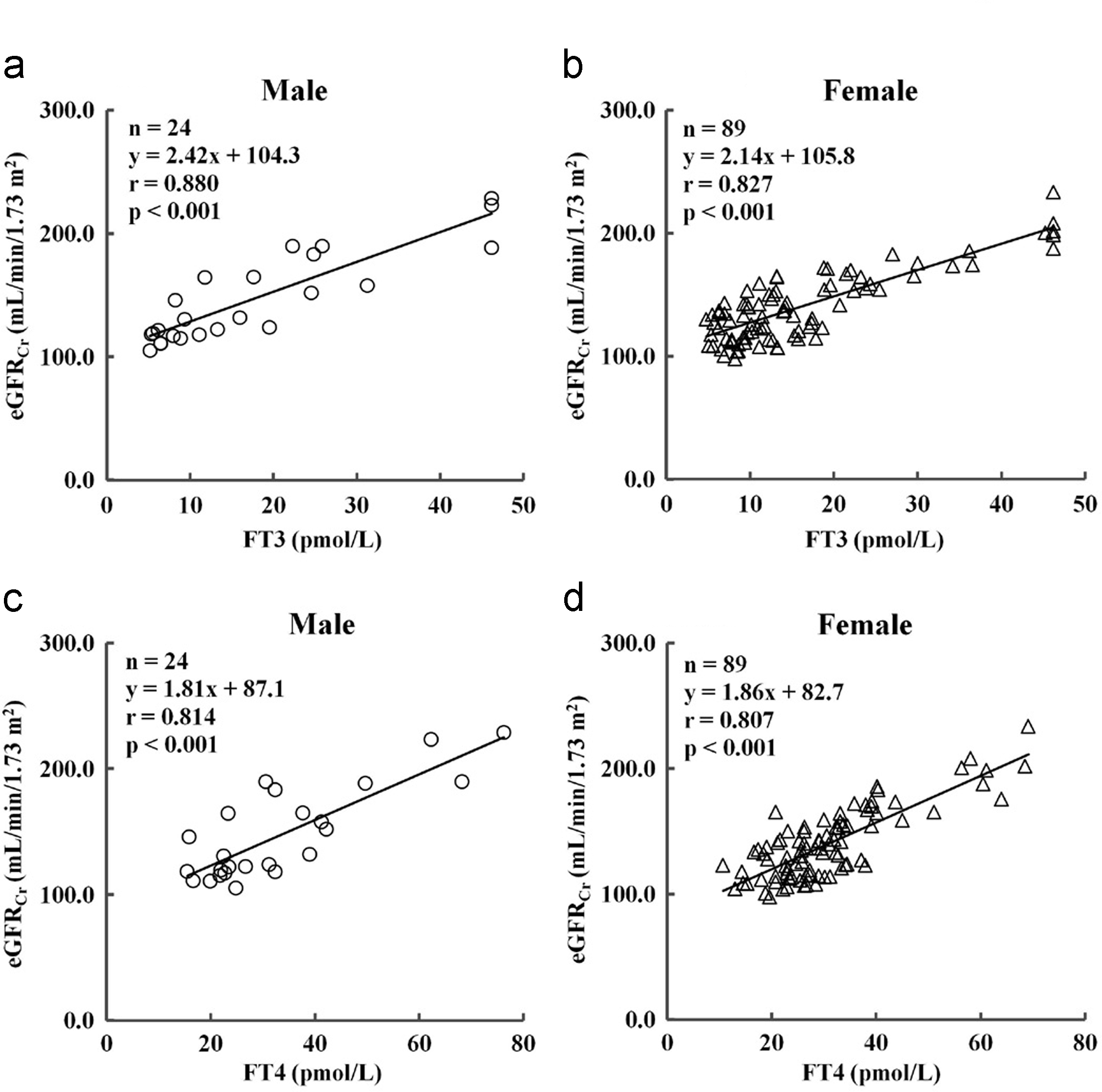

Supplement: Supplemental Fig. 2 — Relationships between serum thyroid hormone levels (FT3 and FT4) and eGFRCr levels in males and females with GD. Estimated glomerular filtration rates (eGFR) were determined from serum creatinine (sCr) levels (eGFRCr) in patients with untreated or poorly controlled GD (n=113). eGFRCr levels in male patients are indicated by circles (○) and in female patients by triangle (△). (a) Relationship between free triiodothyronine (FT3) levels (x-axis) and eGFRCr levels (y-axis) in male patients, (b) relationship FT3 levels (x-axis) and eGFRCr levels (y-axis) in female patients, (c) relationship between free thyroxine (FT4) levels (x-axis) and eGFRCr levels (y-axis) in male patients and (d) relationship between FT4 levels (x-axis) and eGFRCr levels (y-axis) in female patients. [file mmc3.jpg]

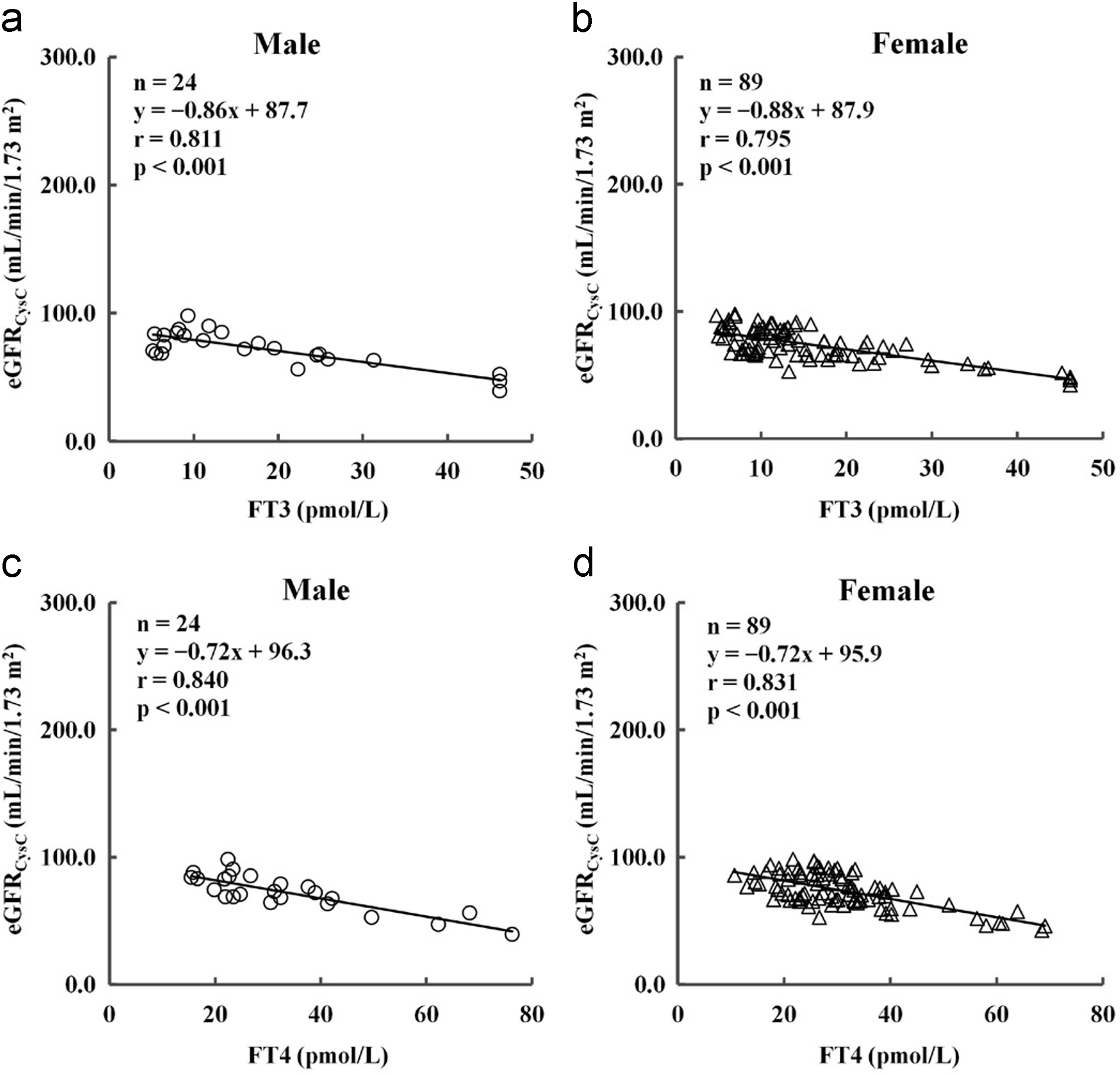

Supplement: Supplemental Fig. 3 — Relationships between serum thyroid hormone levels (FT3 and FT4) and eGFRCysC levels in males and females with GD. Estimated glomerular filtration rates (eGFR) were determined from standardized serum cystatin C (sCysC) levels (eGFRCysC) in patients with untreated or poorly controlled GD (n=113). eGFRCysC levels in male patients are indicated by circles (○) and in female patients by triangle (△). (a) Relationship between free triiodothyronine (FT3) levels (x-axis) and eGFRCysC levels (y-axis) in male patients, (b) relationship FT3 levels (x-axis) and eGFRCysC levels (y-axis) in female patients, (c) relationship between free thyroxine (FT4) levels (x-axis) and eGFRCysC levels (y-axis) in male patients and (d) relationship between FT4 levels (x-axis) and eGFRCysC levels (y-axis) in female patients. [file mmc4.jpg]

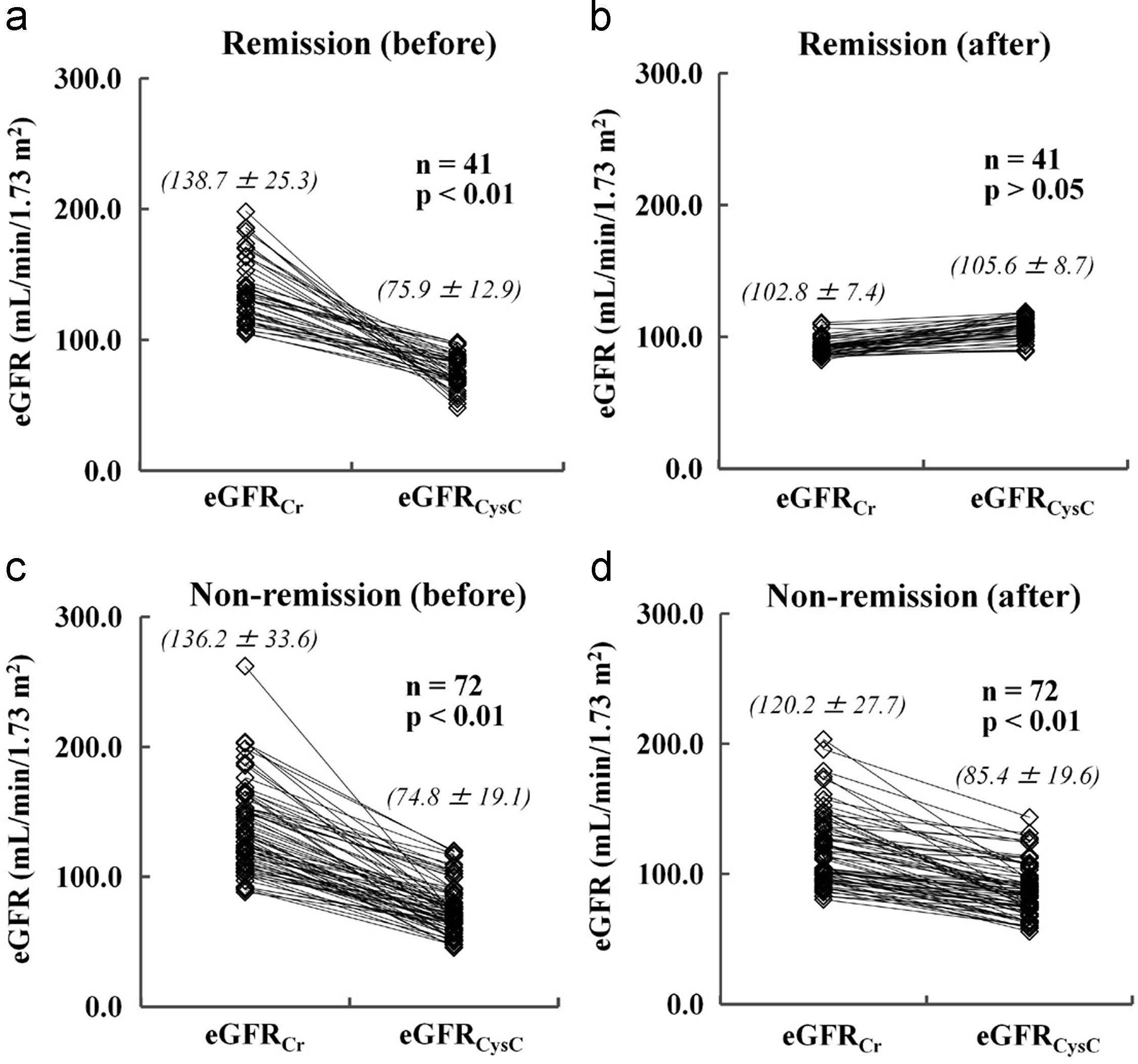

Supplement: Supplemental Fig. 4 — Evaluation of eGFRCr and eGFRCysC levels in patients with GD, before and after treatment, in remission and non-remission groups. Estimated glomerular filtration rates (eGFRs) were determined from serum creatinine (sCr) levels (eGFRCr) and standardized serum cystatin C (sCysC) levels (eGFRCysC) in remission (n=41) and non-remission (n=72) groups. eGFRs are indicated by diamonds (◇). (a) Levels of eGFRCr and eGFRCysC before treatment (remission group), (b) levels of eGFRCr and eGFRCysC after treatment (remission group), (c) levels of eGFRCr and eGFRCysC before treatment (non-remission group) and (d) levels of eGFRCr and eGFRCysC after treatment (non-remission group). [file mmc5.jpg]
